# Comparison Between Ultrasound-Guided Supraclavicular and Interscalene Brachial Plexus Blocks in Patients Undergoing Arthroscopic Shoulder Surgery: A Prospective, Randomized, Parallel Study

**DOI:** 10.1097/MD.0000000000001726

**Published:** 2015-10-09

**Authors:** Taeha Ryu, Byung Tae Kil, Jong Hae Kim

**Affiliations:** From the Department of Anesthesiology and Pain Medicine, School of Medicine, Catholic University of Daegu, Daegu, Republic of Korea.

## Abstract

Although supraclavicular brachial plexus block (SCBPB) was repopularized by the introduction of ultrasound, its usefulness in shoulder surgery has not been widely reported. The objective of this study was to compare motor and sensory blockades, the incidence of side effects, and intraoperative opioid analgesic requirements between SCBPB and interscalene brachial plexus block (ISBPB) in patients undergoing arthroscopic shoulder surgery.

Patients were randomly assigned to 1 of 2 groups (ISBPB group: n = 47; SCBPB group: n = 46). The side effects of the brachial plexus block (Horner's syndrome, hoarseness, and subjective dyspnea), the sensory block score (graded from 0 [no cold sensation] to 100 [intact sensation] using an alcohol swab) for each of the 5 dermatomes (C5–C8 and T1), and the motor block score (graded from 0 [complete paralysis] to 6 [normal muscle force]) for muscle forces corresponding to the radial, ulnar, median, and musculocutaneous nerves were evaluated 20 min after the brachial plexus block. Fentanyl was administered in 50 μg increments when the patients complained of pain that was not relieved by the brachial plexus block.

There were no conversions to general anesthesia due to a failed brachial plexus block. The sensory block scores for the C5 to C8 dermatomes were significantly lower in the ISBPB group. However, the percentage of patients who received fentanyl was comparable between the 2 groups (27.7% [ISBPB group] and 30.4% [SCBPB group], *P* = 0.77). SCBPB produced significantly lower motor block scores for the radial, ulnar, and median nerves than did ISBPB. A significantly higher incidence of Horner's syndrome was observed in the ISBPB group (59.6% [ISBPB group] and 19.6% [SCBPB group], *P* < 0.001). No patient complained of subjective dyspnea.

Despite the weaker degree of sensory blockade provided by SCBPB in comparison to ISBPB, opioid analgesic requirements are similar during arthroscopic shoulder surgery under both brachial plexus blocks. However, SCBPB produces a better motor blockade and a lower incidence of Horner's syndrome than ISBPB.

## INTRODUCTION

Before the introduction of ultrasound to the regional anesthesia field, supraclavicular brachial plexus block (SCBPB) had been abandoned due to the associated high risk of pneumothorax^1–3^ and of inadvertent vascular puncture leading to subsequent systemic local anesthetic toxicity.^4^ Despite its repopularization following the introduction of ultrasound, SCBPB was not used for shoulder arthroscopic surgery because it had been believed that the suprascapular nerve, which innervates 70% of the shoulder joint,^5^ could not be blocked by SCBPB.^6^ Recently, a large prospective study demonstrated the clinical effectiveness of SCBPB for shoulder surgery.^7^ However, approximately 50 mL of a local anesthetic was used in the study,^7^ thereby devaluing the ultrasound guidance, which reduces the local anesthetic requirements for brachial plexus block.^8^ The primary goal of the present study was to compare the sensory and motor blockades, the incidence of side effects, and the intraoperative opioid analgesic requirements between interscalene brachial plexus block (ISBPB), which traditionally has been performed for shoulder arthroscopic surgery,^9^ and SCBPB using 25 mL of local anesthetic.

## METHODS

### Patients

After obtaining approval from the Daegu Catholic University Medical Center Institutional Review Board, Republic of Korea, and written informed consent, this prospective, randomized, parallel group study was conducted using 100 consecutive patients undergoing shoulder arthroscopic surgery under brachial plexus block. This study was registered at http://www.ClinicalTrials.gov (Identifier: NCT01958801). The inclusion criteria were age between 18 and 80 years, American Society of Anesthesiologists physical status I to II, and body mass index <35 kg m^−2^. The exclusion criteria included coagulation deficiency, known allergy to local anesthetics, neurologic deficit on the side of the operation, inflammation at the brachial plexus puncture site, respiratory insufficiency, contralateral hemidiaphragmatic paralysis, pneumonectomy or vocal cord palsy, coronary artery disease, cardiac conduction disorder or arrhythmia, congestive heart failure, diabetes mellitus, hypertension, serum electrolyte abnormality, autonomic dysfunction, psychiatric disorder, patient refusal, and difficulty communicating with medical personnel.

### Randomization and Masking

Eligible patients were equally randomized to receive either ISBPB or SCBPB using random numbers generated by Microsoft Excel 2010 (Microsoft Corp., Redmond, WA). The patients were not informed the details of the local anesthetic injection sites, and the investigators assessed the outcome variables without being involved in the brachial plexus block; therefore, all participants but the anesthesiologist (who performed the brachial plexus block) were blinded to the anesthetic technique.

### Placement of Brachial Plexus Block

**FIGURE 1:**
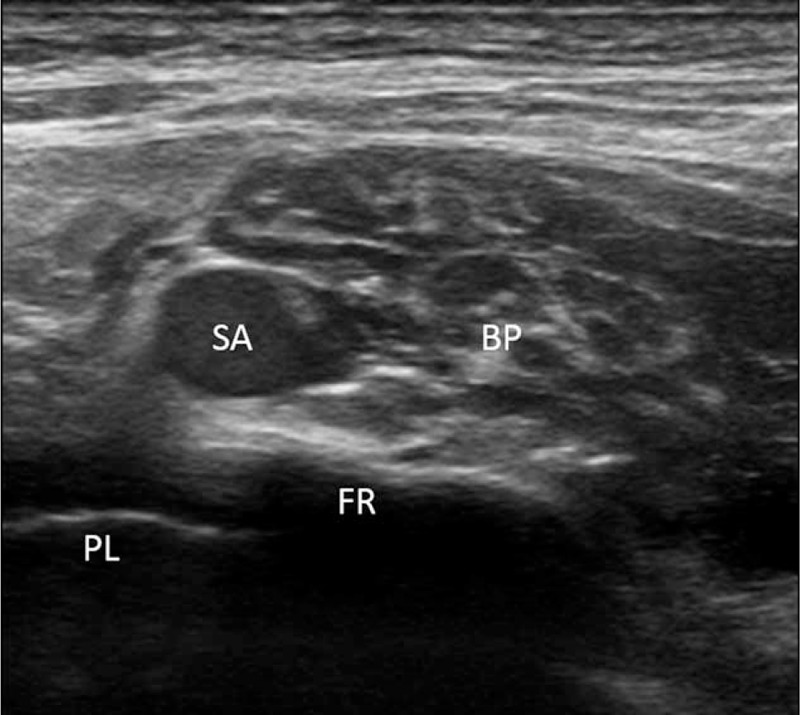
Even distribution of local anesthetics around the brachial plexus located lateral and cephalad to the subclavian artery after supraclavicular brachial plexus block. BP = brachial plexus; FR = first rib; PL = pleura; SA = subclavian artery.

**FIGURE 2:**
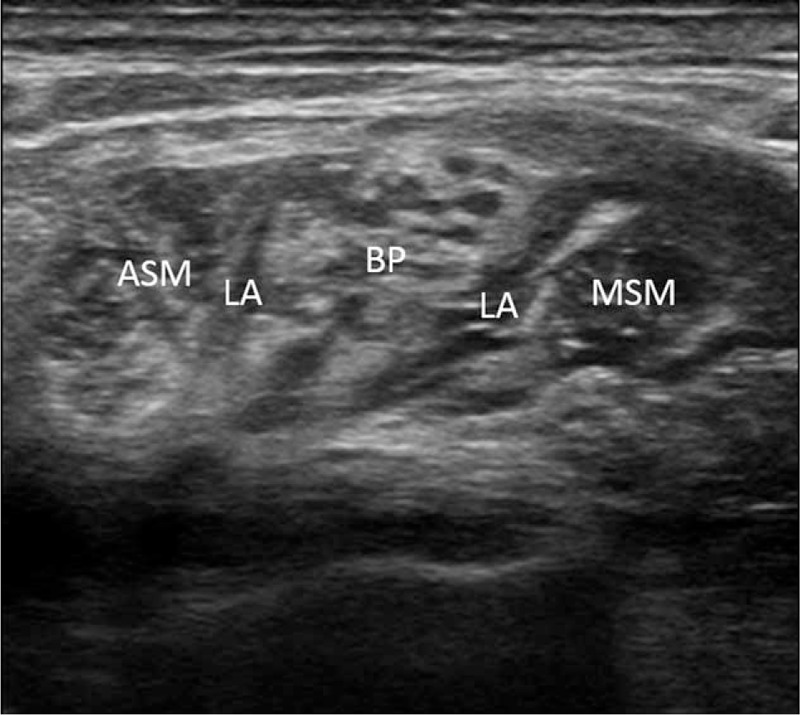
Proximal spread of local anesthetics to the interscalene groove after supraclavicular brachial plexus block. ASM = anterior scalene muscle; BP = brachial plexus; LA = local anesthetics; MSM = middle scalene muscle.

The patients fasted starting at midnight, and a peripheral intravenous infusion of Plasmalyte was started 1 hr before surgery. On arrival to the operating room, electrocardiogram, pulse oximetry, and noninvasive arterial blood pressure monitoring were initiated. The patients were positioned supine with the head slightly rotated to the contralateral side and with the neck extended to facilitate the placement of the ultrasound-guided brachial plexus block. In the ISBPB group, the C5 to C7 or C5 to C8 nerve roots between the anterior scalene and middle scalene muscles were visualized in the absence of the subclavian artery and first rib by placing a 5 to 13 MHz linear phased array transducer (UST-5411, Hitachi Aloka Medical, Ltd., Tokyo, Japan) equipped in a ProSound α7 Premier (Hitachi Aloka Medical, Ltd.) distal to the cricoid cartilage to minimize blocking the phrenic nerve.^10^ Then, a 50-mm, 20-gauge nerve-stimulating needle (Stimuplex^®^ D, B. Braun, Melsungen, Germany) connected to a peripheral nerve stimulator (Stimuplex^®^ Dig RC, B. Braun, Melsungen, Germany) set at 0.2 mA with a stimulating frequency of 1 Hz was placed into the interscalene groove using a lateral-to-medial in-plane technique, and 25 mL of a local anesthetic mixture containing 12.5 mL of 1% mepivacaine and 12.5 mL of 0.75% ropivacaine, for a faster onset and longer duration blockade,^11^ was injected around the nerve roots of the brachial plexus. The needle trajectory was adjusted to facilitate the even distribution of the local anesthetic around each nerve root.^12^ In the SCBPB group, the linear phased array transducer was placed in the supraclavicular fossa to identify distal trunks or proximal divisions of the brachial plexus located lateral and cephalad to the subclavian artery above the first rib. The nerve-stimulating needle was inserted at the lateral border of the linear phased array transducer and advanced until the brachial plexus sheath was penetrated. Once the needle was placed near the brachial plexus, 25 mL of the local anesthetic mixture was injected. The needle was repositioned to surround all of the trunks or divisions of the brachial plexus with the local anesthetic mixture (Figure 1).^8^ At the conclusion of the SCBPB, the transducer was placed proximal to the point where the subclavian artery and first rib became absent, and cephalad spread of the local anesthetics into the interscalene groove was determined (Figure 2). In both groups, under suspicion of intraneural injection based on high resistance to the injection, evoked motor response at a nerve stimulation current of 0.2 mA or less,^13^ or a patient complaint of paresthesia or pain, the injection of the local anesthetics was ceased, and the needle was withdrawn and redirected.

### Assessment of Sensory and Motor Blockade, Side Effects, and Procedural Time

The extent of motor and sensory blockade was evaluated by an anesthesiologist who was not involved in the brachial plexus block 20 min after the injection of the local anesthetics. Using an alcohol swab, the sensory blockade of the C5 to T1 dermatomes of the shoulder^14^ was graded on a scale from 0 (loss of cold sensation) to 100 (intact sensation). The motor blockade was evaluated by rating the muscle contraction forces corresponding to 4 nerves (elbow and wrist extension [radial nerve], finger abduction [ulnar nerve], wrist flexion [median nerve], and elbow flexion [musculocutaneous nerve]) on a scale of 0 to 6 (6: normal muscle force; 5: slightly reduced muscle force; 4: greatly reduced muscle force; 3: slightly impaired mobility; 2: greatly impaired mobility; 1: near complete paralysis; and 0: complete paralysis).^15^ The side effects of the block (Horner's syndrome, hoarseness, and subjective dyspnea, which can be caused by ipsilateral stellate ganglion, recurrent laryngeal nerve, and phrenic nerve block, respectively) and the procedural time (time between insertion and removal of the nerve-stimulating needle) were also recorded.

### Intraoperative Management of Patients

Following completion of the assessments, the patients were placed in a sitting position, and the surgery began. Prior to the creation of a posterolateral portal for initial inspection of the glenohumeral joint, the posterior aspect of the shoulder, which had frequently been spared when performing ISBPB,^12,16–18^ was anesthetized by local infiltration using 10 mL of 1% mepivacaine. Fentanyl was administered in 50 μg increments when the patients complained of pain during the surgery. If an impaired visualization of the surgical field was noted due to bleeding or if a patient's systolic blood pressure increased to above 170 mm Hg during the surgery, hydralazine was administered in 10 mg increments. Vasopressors, inotropes, or chronotropics (eg, ephedrine, epinephrine, atropine) were used at the discretion of the attending anesthesiologist in cases of hypotensive bradycardic events (HBEs), which were defined as intraoperative bradycardia (a decrease in heart rate of more than 30 beats per minute [bpm] in <5 min compared with the baseline heart rate or any decrease in heart rate to <50 bpm at any time after placement in the sitting position) and/or hypotension (a decrease in systolic blood pressure of more than 30 mm Hg in <5 min compared with the baseline systolic blood pressure or any decrease in systolic blood pressure to <90 mm Hg at any time after placement in the sitting position).^19^

### Postoperative Evaluation

Postoperative pain was rated on a numerical scale ranging from 0 (no pain) to 10 (worst imaginable pain) at 1-hr intervals until a patient requested analgesics due to the regression of the block. The type and dose of analgesic agent and its route of administration were at the discretion of the orthopedic surgeon. The duration of surgical anesthesia and postoperative analgesia was defined as the time between the end of the local anesthetic injection for brachial plexus block and the postoperative administration of analgesic agents.

### Statistical Analysis

Based on preliminary observations, we assumed that the difference in sensory block scores for the C5 to C7 dermatomes between patients receiving ISBPB and those receiving SCBPB was 15 with a standard deviation of 25. Allowing for 10% dropout, 100 patients were required to achieve a statistical power of 80% at a 5% significance level (2-tailed). Continuous data were assessed to determine normality using the Kolmogorov–Smirnov and Shapiro–Wilk tests. An assumption of normality was met if any of the null hypotheses of the 2 tests was not rejected. Normally distributed data were expressed as the mean ± standard deviation and were analyzed by an independent-samples Student *t* test. Nonparametric data were expressed as the median (interquartile range) and were analyzed by the Mann–Whitney *U* test. Categorical data were expressed as number of patients (percentage) and were analyzed using either a χ^2^ test or Fisher's exact test (if the expected frequencies in any of the cells of a 2 × 2 contingency table were below 5). A 2-tailed *P* value <0.05 was considered statistically significant. The statistical analysis was performed using IBM SPSS Statistics version 19.0.0 (IBM Corp., Armonk, NY).

## RESULTS

**FIGURE 3:**
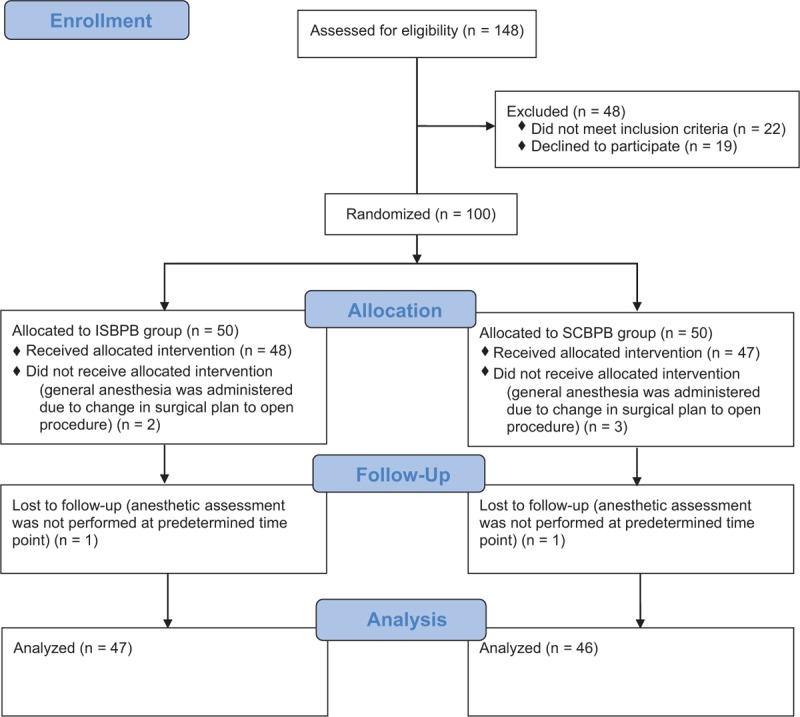
CONSORT diagram. ISBPB = interscalene brachial plexus block; SCBPB = supraclavicular brachial plexus block.

Out of 148 eligible patients, 100 patients participated in the study. Five patients who received general anesthesia due to a change in surgical plan (conversion from an arthroscopic to an open procedure) and 2 patients whose anesthetic assessment could not be performed at a predetermined time point were excluded from the study following randomization (Figure 3). No patient underwent general anesthesia due to pain that was not relieved by brachial plexus block and analgesics. There were no significant differences in the demographic data between the 2 groups (Table 1).

**TABLE 1:**
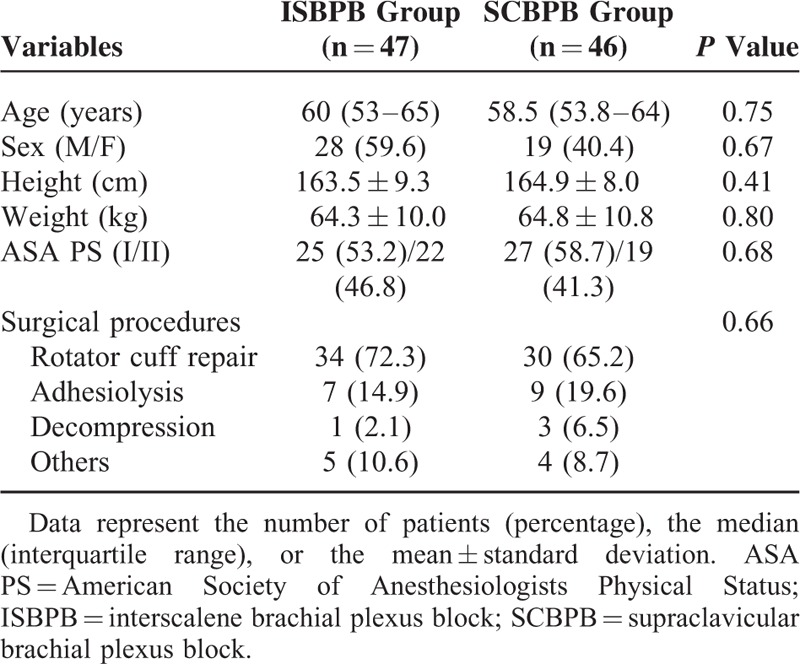
Patient Characteristics

ISBPB led to significantly lower sensory blockade scores for the C5 to C8 dermatomes compared with SCBPB (Table 2). In contrast, significantly lower motor blockade scores of the radial, median, and ulnar nerves were observed in the SCBPB group compared with the ISBPB group (Table 2). Horner's syndrome developed more frequently in the ISBPB group than in the SCBPB group (Table 2). No patients complained of subjective dyspnea. The procedural time was longer in the SCBPB group than in the ISBPB group (Table 2). Proximal spread of the local anesthetics to the interscalene groove was noted in 44 patients (95.7%) in the SCBPB group (Figure 2).

**TABLE 2:**
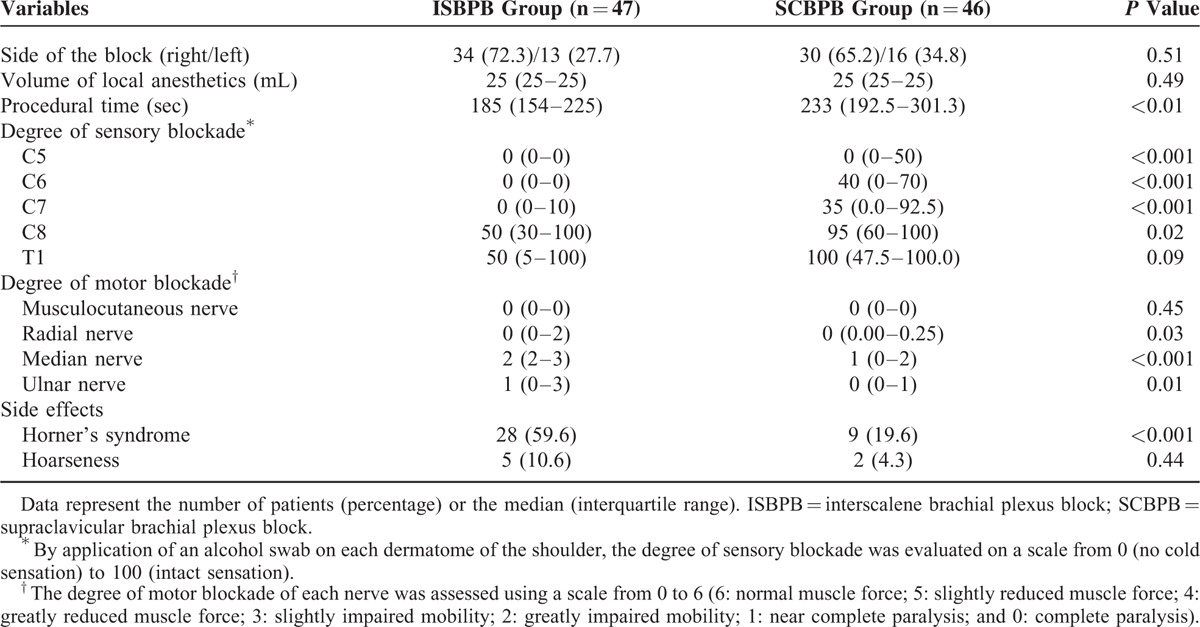
Brachial Plexus Block Characteristics

Fentanyl was administered to 13 patients (27.7%) in the ISBPB group and to 14 patients (30.4%) in the SCBPB group (50 [50–100] μg vs 50 [50–100] μg, *P* = 0.49) (Table 3). Six patients (12.8%) in the ISBPB group and 2 patients (4.3%) in the SCBPB group experienced HBEs (Table 3) (lowest systolic blood pressure: 86.3 ± 11.7 mm Hg; lowest heart rate: 57.3 ± 10.1 bpm) that developed 43.3 ± 27.7 min after the patients assumed the sitting position, and these patients were treated with ephedrine (10 [10–17.5] mg) or atropine (0.5 mg was used in 1 patient). Postoperative analgesic agents were required when a patient's postoperative pain score became 6 (5–6) and 6 (5–6) (*P* = 0.58) at 705 (646.5–831.0) min and 733 (603.5–838.5) min (*P* = 0.66) after ISBPB and SCBPB, respectively (Table 3).

**TABLE 3:**
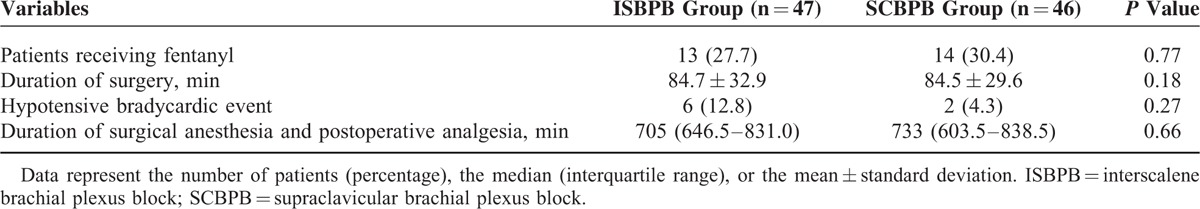
Perioperative Characteristics

## DISCUSSION

We found that SCBPB produced a more intense motor blockade and reduced the incidence of Horner's syndrome compared with ISBPB, although the sensory blockade achieved by SCBPB was inferior to that of ISBPB. Nevertheless, all of the patients underwent surgery with a comparable frequency of opioid use, and none required a conversion to general anesthesia. In addition, both types of brachial plexus blocks resulted in comparable durations of surgical anesthesia and postoperative analgesia, with an insignificant difference in the incidence of HBEs.

Although it provides a rapid and complete block that benefits from the compact topographic arrangement of the brachial plexus trunks, SCBPB had been reluctantly performed in the past due to an associated high incidence of pneumothorax (0.6% to 6%)^1,2^ and, to a lesser extent, inadvertent vascular puncture with resultant local anesthetic toxicity.^4^ However, the introduction of ultrasound to the practice of regional anesthesia has led to a remarkable reduction in these complications.^20^ Despite its emerging popularity, the clinical indications of SCBPB have been confined to upper limb surgeries below the shoulder because of concerns that it is performed too distally from the cervical nerve roots to block the suprascapular nerve,^6^ which innervates 70% of the shoulder joint in addition to the subacromial bursa, coracoclavicular ligament, and acromioclavicular joint.^5^ Therefore, SCBPB had not been commonly used for shoulder surgery until the effectiveness and safety of ultrasound-guided SCBPB for shoulder arthroscopy were demonstrated in a large prospective survey^7^ following a previous report that showed cephalad spread of local anesthetic into the interscalene groove during ultrasound-guided SCBPB.^21^

However, the authors in the above survey used 1.5% mepivacaine with or without 0.75% bupivacaine for both ultrasound-guided ISBPB and SCBPB with a mean volume of 50 mL (range, 20–65 mL).^7^ This volume of local anesthetic is substantially more than the minimum effective volume of 1.5% mepivacaine in 95% of patients for ultrasound-guided SCBPB (17 mL)^8^ and the minimum effective volume of 0.75% ropivacaine for ultrasound-guided ISBPB (5 mL),^22^ both of which were recently determined using a multiple injection technique that spreads local anesthetic more uniformly around the brachial plexus. In addition, the study focused on the postoperative course following surgical anesthesia rather than on the quality of sensory and motor blockades produced by the anesthesia.^7^ Hence, we conducted the present study using 25 mL of a local anesthetic mixture containing 1% mepivacaine and 0.75% ropivacaine with subsequent assessment of sensory and motor blockades to address the issues raised above.

Because SCBPB blocks the brachial plexus at the level of the distal trunks or proximal divisions, it is considered to spare the suprascapular nerve,^6^ which is described to arise from the upper trunk proper according to most anatomy books.^23,24^ However, this assumption is challenged by a case that showed a “chimney effect,” which forced local anesthetic to spread up between the anterior and middle scalene muscles^21^ and a recent anatomical study that reported that the suprascapular nerve originates from a posterior division of the upper trunk 3.1 ± 3.1 mm distal to the bifurcation of the upper trunk or the point of upper trunk bifurcation in 90% of 100 cadaveric subjects.^25^ Based on this evidence, SCBPB performed at the distal trunk/proximal division level is sufficient to block the suprascapular nerve, which primarily originates from or is distal to the upper trunk bifurcation. Even if its origin is the upper trunk (6%) or C5 nerve root (4%),^25^ the suprascapular nerve might be blocked by local anesthetic that spreads cephalad from the site of injection. In the present study, 95.7% of the SCBPB group exhibited local anesthetic spread into the interscalene groove, and all of the patients in the SCBPB group underwent surgery without conversion to general anesthesia. Therefore, the traditional indications for SCBPB (surgery of the hand and arm)^26^ must be expanded to shoulder surgeries.

In this study, sensory blockade of the C5 to C8 dermatomes in the shoulder region was inferior in the SCBPB group compared with the ISBPB group (Table 2). These findings did not confirm our expectation that SCBPB would provide more effective analgesia in the posterior aspect of the shoulder region (C8 and T1 dermatomes), which often remains incompletely blocked by classic ISBPB^12,16–18^ or ultrasound-guided ISBPB^12^; ultrasound-guided SCBPB feasibly enables visualization of the inferior trunk forming the C8 to T1 division,^27^ which is difficult to visualize at the nerve root level by ultrasound due to its deep and caudal location.^28^ In contrast, the motor blockade (radial, median, and ulnar nerves) that was assessed below the shoulder was more intense in the SCBPB group than in the ISBPB group (Table 2), presumably due to ulnar sparing associated with ISBPB. Hence, it is speculated that SCBPB provides a more potent blockade distal to the shoulder, whereas local anesthetics travelling to the interscalene groove do not offer the same extent of blockade of the shoulder, which is provided by ISBPB. In a previous study, nerve stimulator-guided SCBPB achieved a significantly higher rate of complete sensory and motor blockade than did nerve stimulator-guided ISBPB.^29^ However, it is uncertain whether the sensory blockade was assessed in the shoulder region.

Horner's syndrome, which develops following ISBPB and SCBPB with a varying incidence of 1% to 75%,^16,30–32^ resolves in concert with local anesthetic block resolution, and this syndrome has been considered to have no clinical relevance.^33^ However, Horner's syndrome was recently suggested to be a contributing factor for HBEs that occur in patients undergoing shoulder surgery in the sitting position.^8^ Therefore, efforts to reduce the incidence of HBEs are necessary. In this respect, the introduction of ultrasound to the practice of regional anesthesia seems to be promising based on the reduction in the incidence of HBEs in patients undergoing ultrasound-guided techniques (5% in ISBPB^30^ and 1% in SCBPB^31^) compared with those undergoing conventional techniques (75% in ISBPB^16^ and 64.1% in SCBPB^32^). Consistent with the results of a previous study, which showed that the incidence of Horner's syndrome was lower after SCBPB using nerve stimulation than after ISBPB using nerve stimulation,^29^ the SCBPB group experienced Horner's syndrome less frequently than the ISBPB group (Table 2) in the present study. However, an insignificant difference in the incidence of HBEs between the 2 groups precluded drawing meaningful conclusions regarding the beneficial effects of the reduced incidence of Horner's syndrome in the SCBPB group based on the occurrence of HBEs.

Compared with nerve stimulation alone, ultrasound with or without nerve stimulation enables the accurate placement of local anesthetic around peripheral nerves and reduces the incidence of vascular puncture, thereby leading to a reduction in the rate of analgesic rescue.^34^ However, there was no significant difference in the frequency of rescue analgesic administration for ultrasound guidance with or without nerve stimulation.^34^ Furthermore, the addition of nerve stimulation to ultrasound guidance required a long procedural time without any beneficial effect on the success rate of the ultrasound-guided block.^35–38^ Nevertheless, nerve stimulation was used in combination with ultrasound in the present study because ultrasound guidance alone^39^ or the combination of the 2 guidance techniques^40^ cannot completely prevent intraneural injections that cause neurologic complications.

There are several limitations to be considered in this study. Although the sensory blockade was assessed at the shoulder level, the assessment of the motor blockade could not be performed at the shoulder level due to inconsistencies in shoulder pathology among the patients (Table 1), which precluded the application of a uniform physical examination. In addition, due to overlapping cutaneous sensory innervation to the cape of the shoulder,^14,26^ the sensory blockade of the supraclavicular nerves, which are branches of the superficial cervical plexus (C3–C4), could not be distinguished from the dermatomal blockade of the brachial plexus. Despite the inferior sensory blockade in the SCBPB group (Table 2), the number of patients who required opioid analgesia was comparable between the 2 groups (Table 3). These contradictory results might have arisen from the short interval (20 min) between the sensory blockade assessment and the end of the local anesthetic injection compared with the minimum interval (50 min) that is required to attain a maximal blockade.^15^ Lastly, although no patients complained of subjective dyspnea under the present study protocol, in which ISBPB was performed at lower levels beneath the cricoid cartilage, ipsilateral hemidiaphragmatic paresis resulting from phrenic nerve block could not be ruled out in this study.

Although a less potent sensory blockade was provided by SCBPB compared with ISBPB, a better motor blockade profile and lower incidence of Horner's syndrome might be obtained when using SCBPB. Despite the discrepancies in the sensory blockade assessment in the early postblock period, neither of the 2 brachial plexus blocks required conversion to general anesthesia due to inadequate sensory anesthesia. In addition, the patients receiving either SCBPB or ISBPB required opioid analgesics with a comparable frequency and benefited from similar surgical anesthesia and postoperative analgesia. In conclusion, SCBPB can be performed as an alternative to ISBPB in patients undergoing arthroscopic shoulder surgery.
